# Acute generalized exanthematous pustulosis simulating toxic epidermal necrolysis: case presentation and literature review

**DOI:** 10.1186/s13223-020-0407-5

**Published:** 2020-02-04

**Authors:** Ana-Maria Copaescu, Danielle Bouffard, Marie-Soleil Masse

**Affiliations:** 1 Allergy and Immunology Department, Centre Hospitalier de l’Université de Montréal (CHUM), 264 Boulevard René-Lévesque E, Montréal, QC H2X 1P1 Canada; 2 Pathology Department, Centre Hospitalier de l’Université de Montréal (CHUM), Montréal, Canada

**Keywords:** Acute generalized exanthematous pustulosis, Toxic epidermal necrolysis, Severe cutaneous adverse drug reaction, Disease overlap, Beta-lactam antibiotics

## Abstract

**Background:**

Acute generalized exanthematous pustulosis (AGEP) and toxic epidermal necrolysis (TEN) are severe cutaneous adverse reactions. These rare conditions differ in clinical presentation, pathological features, treatment and prognosis, but overlap has been described implying a challenging clinical management.

**Case presentation:**

We describe a case of overlap between TEN and AGEP probably secondary to beta-lactams in a 77-year-old patient treated for a complicated cholangitis. We review the diagnosis and the management of these two conditions. The diagnosis of TEN was suggested by the initial clinical presentation with severe hemodynamic instability, skin detachment, positive Nikolsky sign and mucosal involvement. However, the skin biopsy as well as the rapid improvement of the skin lesions were discriminative for AGEP. This indicated an overlap presentation. Unfortunately, the patient refused allergy investigations in order to find the culprit drug. Medical photographs, proper physical examination and histopathological results are integrated.

**Conclusion:**

Despite clinical features indicating a diagnosis of TEN, histopathology was conclusive for AGEP thus indicating a possible clinical-pathological overlap between the two conditions, a scarcely described situation in the medical literature. To our knowledge, this is one of the few cases that portrays a TEN–AGEP overlap probably secondary to Piperacillin Tazobactam. Understanding the immunological implications of these conditions can help us better distinguish and manage these severe reactions.

## Background

A severe adverse drug reaction is defined as a life threatening response that requires inpatient care or results in persistent disability or incapacity [1]. Toxic epidermal necrolysis (TEN) and acute generalized exanthematous pustulosis (AGEP) are examples of severe adverse cutaneous drug reactions. These conditions differ in clinical manifestations, morphological features, prognosis and treatment, but severe cases of AGEP rarely mimic TEN. The literature describes thirteen cases where the clinical presentation represented a challenge to patient care. The main causal drugs are antibiotics, nonsteroidal anti-inflammatory drugs (NSAIDs), narcotics (morphine), anti-epileptics (carbamazepine), hydroxychloroquine and chemotherapy drugs.

In this report, we describe the case of a 77-year-old female who developed an unusual AGEP–TEN overlap probably secondary to Piperacillin Tazobactam. The patient presented with severe skin detachment, a positive Nikolsky sign, hemodynamic instability and was hospitalized in the burn unit. However, the biopsy was consistent with AGEP. We offer a global portrait of this condition including the initial clinical description, the treatment options and the patient’s clinical evolution. A discussion and a literature review will follow.

## Case presentation

A 77-year-old female with no known allergies and with a medical history including dyslipidemia, hypertension, hypothyroidism was transferred to our intensive care unit from a community hospital for an important skin eruption. Prior to her transfer, she had been hospitalized for approximately 1 month for cholangitis complicated by sepsis, iatrogenic pancreatitis and portal vein thrombosis. After a chart review, it was noted that she had received Piperacillin Tazobactam for a total of 20 days as well as Ibuprofen as needed. She also had two contrast radiologic exams 5 and 21 days prior to the eruption.

**Fig. 1:**
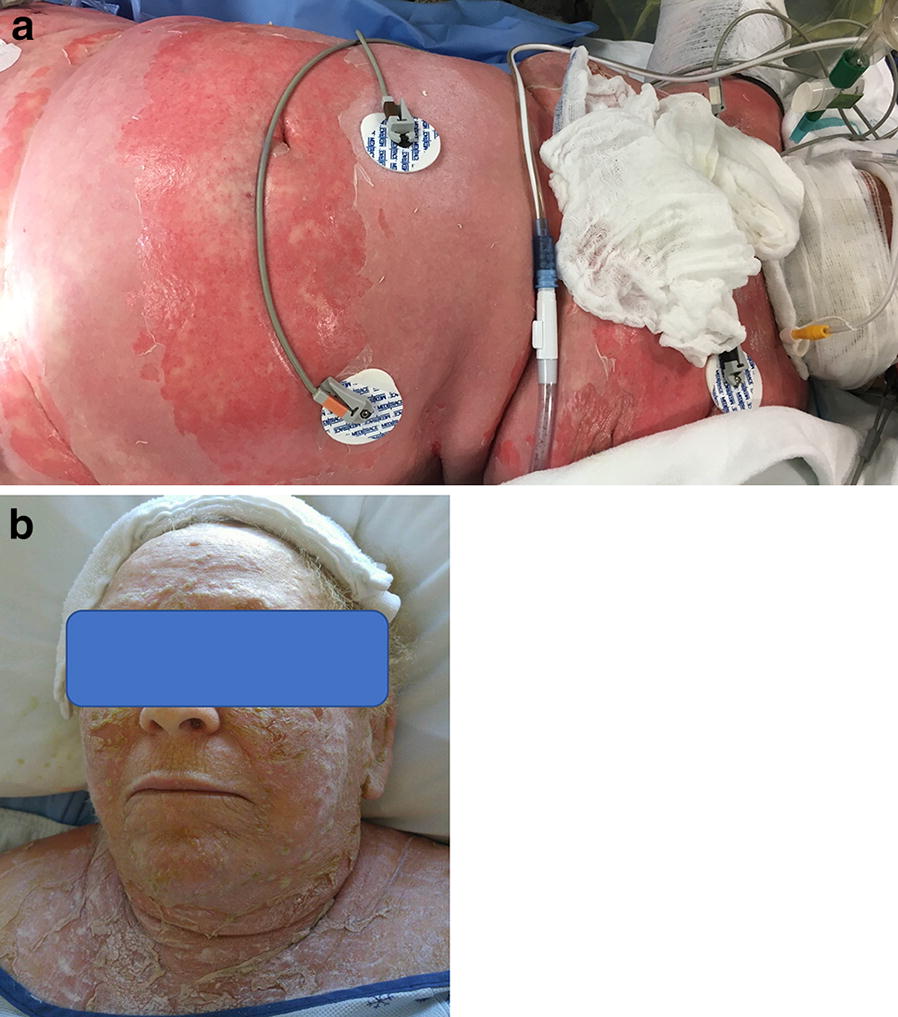
Clinical photographs. **a** Confluent erythematous and edematous plaques with peeling affecting 35% and a positive Nikolsky sign. **b** Severe peeling after resolution of blistering lesions on the patient’s face

On initial evaluation, the patient was intubated in the context of an important vasoplegic shock with acute kidney failure. She presented with erythema and skin detachment on 80% and 35% of her total body surface, respectively, including external genital area but no ophtalmic lesions (Fig. 1). She presented multiple bullae with purulent liquid. With these findings as well as a positive Nikolsky sign on her arms, abdomen and neck region, the most probable diagnosis was toxic epidermal necrolysis (TEN). Her TEN score (Table 1) was calculated at 3, indicating a very high mortality risk. Twenty-four hours after admission, the patient was sub-febrile (body temperature increased to 38.2 Celsius) and her leukocyte level reached 16.8 × 10^9^/L.

**Table 1:** SCORTEN scale (reproduced from Bastuji-Garin et al. [28])

Risk factors
Age (> 40 years)
Associated malignancy
Heart rate (> 120 beats/min)
Serum BUN (> 10 mmol/L)
Detached or compromised body surface (> 10%)
Serum bicarbonate (< 20 mEq/L)
Serum glucose (> 14 mmol/L)

The number of risk factors is correlated with the mortality rate (i.e. for one or less risk factors, the calculated mortality rate is 3.2%, for 2 risk factors, the mortality is 12.1%, for 3, 35.3%, for 4, 58.3% and for 5 and more, the mortality rate is more than 90%

**Fig. 2:**
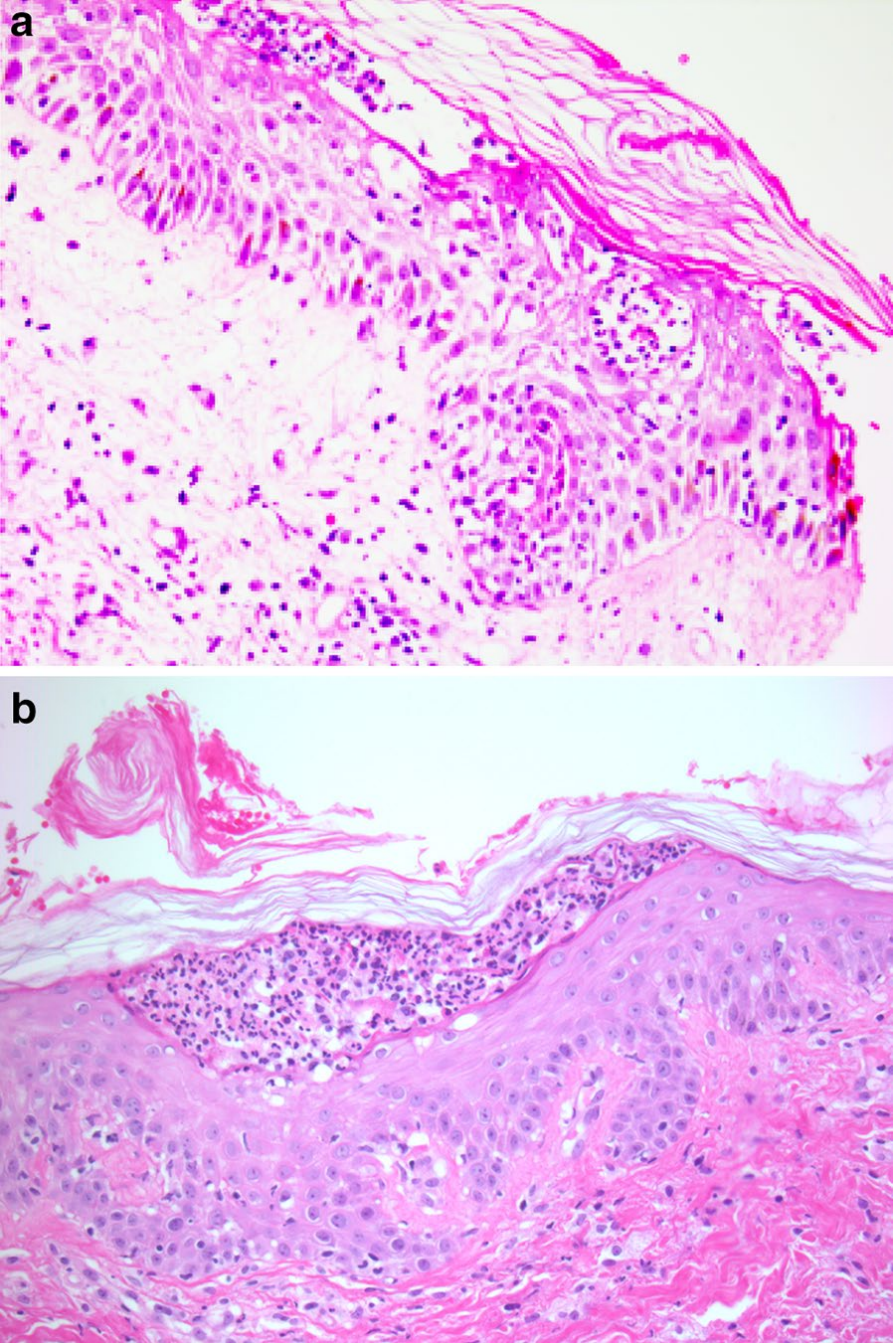
Histologic picture. **a** This biospy is from the hand and shows subcorneal pustules with epidermal spongiosis and papillary derma edema. Note the presence of necrosis in the intraepidermal portion of a sweat duct at the edge (hematoxylin and eosin, ×20). **b** This biopsy is from the thigh and shows a subcorneal pustule (hematoxylin and eosin, ×20)

She was treated with intravenous immunoglobulins 1 g/kg/day for a total of 3 days. A skin biopsy from the hand and one from the thigh were obtained upon admission. These biopsies showed sub-corneal pustules with edema of the papillary dermis and an inflammatory infiltrate containing eosinophils. Scattered necrotic keratinocytes were described, but there was no confluent epidermal necrosis. Necrotic keratinocytes were also noted in the intraepidermal portion of a sweat gland (Fig. 2). Direct immunofluorescence was negative. Therefore, the final histopathological diagnosis was compatible with acute generalized exanthematous pustulosis (AGEP).

Several agents were identified as possible culprit: b-lactam antibiotics (Piperacillin Tazobactam), radiologic iodine product and anti-inflammatory drugs (Ibuprofen).

On the 3rd day after admission, she was extubated. After 7 days, no new lesions were noted and the desquamation resumed to less than 5% of the total body surface. She had an excellent evolution and she was eventually transferred to the referring hospital for rehabilitation.

At 2 months follow-up, the patient was doing well and her skin had healed leaving no scarring. She had the medic-alert bracelet indicating the above-mentioned drugs. Unfortunately, the patient refused allergy investigations such as patch testing in order to confirm the culprit drug.

## Discussion and conclusions

### Better understanding AGEP and TEN

Both AGEP and TEN are classified as drug reactions. Each clinical entity is the result of an intricate connexion between genetic, immunologic and environmental factors. Their immunological mechanisms are not completely understood but it is well defined that hey both are T-cell-mediated type IV hypersensitivity reactions as portrayed by Gell and Coombs. With increased understanding of the difference between the hypersensitivity reactions included in this group, a sub-classification was described according to the pattern of cytokine production by different T cells and the contribution of certain types of leucocytes. Thus, TEN is a type IVc cytotoxic reaction involving CD8+ cytotoxic killer cells and AGEP is a type IVd reaction that is mainly characterized by the production of neutrophil-attracting chemokine [2]. Research is ongoing to characterize specific cytokines that could play a crucial role in the pathophysiology of AGEP and TEN.

These conditions have very different clinical characteristics. To begin with, the period from starting the culprit drug and the beginning of the skin eruption is about 2 to 3 weeks for TEN, while some AGEP eruptions may begin after a 24-h latency period. The morphological description varies significantly, AGEP being characterised by small pustules on an erythematous background and TEN being recognized by the presence of target lesions, vesicles, bullae, skin detachment with a positive Nikolsky sign and mucosal involvement. In AGEP patients, some authors [3] have described a pseudo positive Nikolsky sign that could represent the coalescence of multiple pustules.

In atypical cases, the biopsy is decisive to formulate the right diagnosis. In our case, the pathologist described subcorneal pustules and edema of the papillary dermis, consistent with AGEP. There have been cases of histological overlap with specific markers of each condition described in the literature [4]. This is not the case of our patient as the pathological features were discriminative for AGEP.

The course of AGEP is usually benign and this condition usually improves after discontinuing the culprit drug. We know that this is not the case for TEN, which is considered life-threatening and can induce multiple long-term complications. These differences and many others are summarized in Table 2.

**Table 2:** Description of AGEP and TEN

	AGEP	TEN
Incidence	1–5/million/year	2–7/million/year
Etiology	Drug (90%) viral, bacterial, or parasitic infections spider bites	Drug (60%) *M. pneumoniae* infections 1/3 cases no cause
Clinical presentation		
Distribution pattern	Intertriginous (generalized)	Generalized
Mucous Membrane	20% (oral)	100% (> 30%)
Pustules	Yes	No
Target lesions	No	Yes
Nikolsky sign	Rare	Yes
Fever	Yes	Yes
Timing	Hours–days	Days–weeks (< 8 wks)
Clinical course	Resolution/re-epithelialization 2-4 weeks	
Histological features	Spongiform subcorneal and/or intraepidermal pustules edema of the dermis, necrosis of single keratinocytes, and an inflammatory infiltrate of neutrophils and eosinophils with perivascular accentuation	Keratinocyte necrosis (partial to full-thickness necrosis of all epidermis layers) perivascular, discrete lymphohistiocytic, inflammatory infiltrate (some eosinophils) in the superficial dermis, ± subepidermal bullae
Prognosis (mortality)	Resolution 2–4 weeks	Acute phase 8–12 days Mortality 30%
Treatment	d/c drug	d/c drug PO or IV corticosteroids, IV immunoglobulin, cyclosporin, anti-TNF

An interesting observation was formulated by Meiss et al. [5] relating that similar cases of overlap might actually be a two-phase clinical reaction pattern, thus a progression from an AGEP with classic pustules to systemic clinical manifestations characteristic of TEN. Unfortunately, our patient was hospitalized in another center before her hemodynamic instability and thus a complete physical exam before admission is lacking.

A very interesting recent article retrospectively studied Steven-Johnson syndrome/TEN mimickers from four academic hospitals including 208 patients [6]. Out of these patients, 13 (6.2%) had a revised diagnosis of AGEP. The authors concluded that the presence of an atypical target lesion, a positive Nikolsky sign, fever and lymphopenia help predict SJS/TEN.

As described, our patient had a positive Nikolsky sign and was subfebrile. However, no atypical target lesions were described and lymphopenia was absent.

### AGEP and TEN overlap—literature review

As mentioned, both AGEP and TEN are rare skin conditions. Combining both conditions in a patient, either because of the clinical manifestations or the histopathological features is even more rare and we found 21 cases described in the literature. In Table 3, we summarize these different cases. It can be noted that there is no tendency towards a specific age group as the patients portrayed are either young adults, middle aged or geriatric patients. There is a slight female predominance in the cases described (14 females and seven males).

**Table 3:** AGEP and TEN overlap cases—literature review

Source	Patient	Culprit drug	Clinical description	Time onset	Biopsy	Treatment	Outcome
Cohen et al. [16]	M 91	Cefuroxime Paracetamol	Generalized erythema (95%), non follicular pustules, bullae (clear) Skin detachment (41%) Nikolsky+	10 days after drug initiation	Spongiform pustules, papillary edema, perivascular mononuclear infiltrate	d/c drug Wet dressing	Resolution after 10 days
Scheinfeld et al. [17]	F 60	Famotidine	Generalized erythema, pustules Erosions Nikolsky+	2 days after drug initiation	Subcorneal blistering, no necrotic keratinocytes	d/c drug Clobetasol propionate	Resolution after 3 days
Byerly et al. [18]	F 45	Valdecoxib	Generalized erythema, nonfollicular Pustular papules and plaques; 80% BSA Nikolsky−	5 days after drug initiation	Spongiform pustules, neutrophilic and eosinophilic infiltrate	d/c drug Wet dressing (bacitracin) IV fluids	n/a
Meiss et al. [5]	M 34 M 49 M 43	Ampilicillin, sulfabactam Clindamycin Amoxicillin	Edematous erythema Oinhead-sized pustules, bullae formation + exfoliation Nikolsky+	n/a	M 49-keratinocytes necrosis of basal layer, neutrophils with subcorneal pustule formation	d/c drug Infliximab	Resolution after 6-14 days
Goh et al. 2008 [4]	F 28	Carbamazepine	Generalized erythema Non-follicular pustules + clear bullae; 55% BSA, Mq membrane Nikolsky+	14 days after drug initiation	Mild spongiosis, subcorneal pustule, necrosis of the epidermis	d/c drug IV Hydrocortisone IV Ig	Resolution after 9 days Minimal scarring
Lateef et al. [19]	F 67—SLE	Hydroxy-chloroquine	Generalized erythema Entire BSA, targetoid patches Conjunctivitis Extensive desquamation	19 days after drug initiation	Epidermal spongiosis, intradermal infiltrate of neutrophils	d/c drug, IV fluids IV Hydrocortisone IV Ig	Resolution after 16 days Minimal scarring
Sadighha et al. [15]	F 56	Amoxicillin/clavulanic acid	Pustules, vesicles, blisters, erythema multiforme-like lesions Oral + conjunctival inflammation	3 days after drug initiation	Keratinocyte necrosis of the basal layer	d/c drug IV fluids Etanercept s/c	Resolution after 18 days
Peermohamed et al. [20]	M 20	Piperacillin and tazobactam	Diffuse erythema, nonfollicular pustules vesicles and bullae Nikolsky+	1 days after drug initiation	Intraepidermal pustules, bulla formation, no necrosis	d/c drug IV Hydrocortisone IV Ig	Resolution after 14 days No scarring
Kardaun et al. [21]	F 70	Morphine	Generalized erythematous eruption Flexure Tiny pustules Superficial erosions	8 days after drug initiation	Spongiform subcorneal pustules, neutrophilic spongiose, few necrotic keratinocytes	d/c drug	Resolution after 14 days
Natkunarajah et al. [14]	F 19	Flucloxacillin	Pin-head pustules on confluent erythema Vesicles, bullae Nikolsky+	5 days after drug initiation	Subcorneal pustule	d/c drug Ciclosporin PO	Resolution after 7 days
Hattem et al. [22]	F 30	Flucloxacillin	Generalized erythema, pustules Large bullae + erosions Nikolsky+	5 days after drug initiation	Spongiform subcorneal pustules + neutrophils, dermal edema	d/c drug IV fluids IV Hydrocortisone	Resolution after 10 days
Moling et al. [23]	M 29	Amoxicillin/clavulanic acid	Severe mucositis and pustules Nikolsky+	5 days after drug initiation	n/a	d/c drug IV methyl- prednisolone	Hopital discharge after 5 days
Kiyohara et al. [24]	M 37	Lamotrigine	Vesicles, pustules, bullae on erythematous base Nikolsky+	14 days after drug initiation	Subcorneal pustules, interface dermatitis	d/c drug Prednisolone PO	Resolution after 10 days
Smith et al. [11]	F 36	Flucloxacillin	Generalized erythema (90%), pustules, bullae Nikolsky+	n/a	Multiple subcorneal pustules, epidermal necrosis	d/c drug	n/a Full recovery
Worsnop et al. [25]	F 23	Flucloxacillin	Generalized erythema, painful blisters Nikolsky+	14 days after drug course	Papillary dermal edema, subepidermal bullae, subcorneal pustule	d/c drug IV Ig IV methyl-prednisolone	n/a Skin healed
Horcajada-Reales et al. [26]	F 87	Amlodipine or Furosemide	Erythematous plaques—violaceous center, detachment, perilabial Nikolsky+	7 days after drug initiation	Subcorneal plaques, perivascular dermal infiltrate	d/c drug IV Ig IV corticosteroids	Resolution after 14 days
Horcajada-Reales et al. [26]	F 75	Teicoplanin Amlodipine	Papulopustular exanthema trunk + limbs Epidermal detachment (30%), labial mucosa Nikolsky+	1 days after drug initiation	Pustules, spongiose, dense superficial perivascular inflammatory infiltrate	d/c drug IV corticosteroids	Resolution after 3 days
García Abellán et al. [27]	F 82	Trimethoprim Sulfamethoxazole	Violaceous exanthema Skin detachment Nikolsky+	14 days after drug initiation	Subcorneal pustules, neutrophils	d/c drug Prednisone PO IV Ig	Resolution after 8 days
Moreno-Arrones et al. [3]	F 90	Vismodegib	Generalized erythema Plaques with purpuric centers—head, folds, and trunk Skin detachment 15% Nikolsky +	8 days after drug initiation	Neutrophilic spongiotic pustules without epidermal necrosis	d/c drug, Ciclosporin	Resolution after 14 days

*M* male, *F* female, *d* days, *d/c* discontinuation, *n/a* not available, *IV* intravenous, *s/c* subcutaneous, *PO* by mouth, *Ig* immunoglobulins

As for the culprit drugs questioned, there are several classes of medications but the antibiotics tend to be suspected more frequently with flucloxacillin, a penicillin beta-lactam antibiotic, being on top of the list. In terms of clinical presentation, some cases initially displayed pustules characteristic of AGEP but these skin lesions evolved towards vesicles, bullae and skin detachment with a positive Nikolski sign in a majority of cases. Thus, the patients presented clinical manifestations of TEN but the histopathological examination favored AGEP, with subcorneal spongiform pustules. The clinical evolution and prognosis were more consistent with an AGEP with patients mostly recovering in the first 2 weeks with no residual scarring.

The hemodynamic instability is a feature rarely described in AGEP. Nevertheless, some authors [7–9] have detailed severe atypical forms of AGEP that presented with systemic inflammatory responses and extensive organ involvement. This form of AGEP might be more frequent in elderly patients with comorbidities. Although some systemic involvement was described in both AGEP and TEN, the presence of extensive skin detachment requiring intensive care admission and support care is more typical of TEN.

### Investigations

Testing for the causal agent in severe drug reactions remains an area of controversy and the management diverges largely among different regions in the world. Intradermal or patch testing varies in terms of availability, drug concentrations and the use of oral challenges [10]. However, the current literature supports using patch testing in certain specific phenotypes. The method is considered safe with minimal risk of systemic reactions and its sensitivity depends on the culprit drug and the type of non-immediate reaction. Despite the benefits of patch testing in identifying the causal drug, only few articles provide a description of this investigation in cases of overlap [11]. Thus, it can be hypothesised that even though physicians might consider patch testing in classic AGEP patients, the severity of an overlap with clinical features of TEN might discourage the medical team to use this investigational tool. Furthermore, the results from patch testing are intended to help the clinician carry out drug challenges to the negative skin tests results. This is scarcely portrayed.

This would have been our approach for this patient if she would have accepted the allergy investigations.

However, novel methods of investigation are on the way. A study from Thailand [12] underlined the importance of detecting drug-specific IFN-y-releasing cells that could help identify up to 46% of causing agents for SJS/TEN and up to 31.3% for AGEP.

### Management

Because of the rarity of these severe skin conditions, management is still a source of discussions and research. Authors agree that the first most important step is the withdrawal of the suspected drug and correct identification of the adverse reaction. In patients diagnosed with AGEP, a resolution of the skin eruption is expected within several days after discontinuation of the drug.

In TEN patients, another critical management step is the supportive care fundamental for every burn or intensive care unit patient that presents with severe skin wounds, hemodynamic instability and/or organ failure. Skin care is currently center dependent and there is no evidence to favor the use of debridement, one dressing over another or the correct method to maintain fluid balance [13].

Another favored treatment is the use of corticosteroids as can be observed in the cases of overlap described in the literature. However, their use is still controversial for TEN and data on survival advantage is contradicting. As for AGEP, even if research is further needed, the use of corticosteroids was correlated with a decrease in hospitalization time.

Intravenous immunoglobulins showed no clear benefit in the studies, but no severe side effects either, so clinicians tent to use them quite often for TEN. This is not part of the management for AGEP.

Other case reports [3, 14] mention a good clinical response to cyclosporin with rapid resolution of skin eruption.

Meiss [5] has described three cases suggesting an overlap or a two-phase clinical reaction of AGEP and TEN. These patients had high serum tumor necrosis factor (TNF) levels and they responded successfully to a TNF-inhibitor, Infliximab. Further studies are needed to confirm that this success story indicates similar physiopathology in these two conditions.

Another TNF-inhibitor, Etanercept, was used by Sadighha [15] in an overlap case with remarkable results observed only hours after the initial dose as well as rapid cessation of progression of the cutaneous lesions. This author underlines the immunological benefits of using this agent as it decreases a number of cells and cytokines that might play a crucial role in these conditions.

### Conclusion

The initial diagnosis of these skin conditions is based on the clinical presentation. Distinguishing AGEP from TEN allows prompt evaluation and accurate treatment. Our patient presented with severe skin detachment and a positive Nikolsky sign, hallmarks of SJS-TEN. Further pathological investigations and the overall clinical evolution oriented us towards an AGEP diagnosis.

We must ask ourselves if these conditions should actually be described as an overlap presentation or as the manifestation of a severe, aggressive AGEP because of the convincing biopsy?

Further case descriptions and systematic research are need to help us elucidate if these atypical cases are an overlap of the two conditions, a two-phase clinical disease entity of a manifestation of severe AGEP mimicking TEN. We thus encourage clinicians to describe these cases and we believe that an international register of these conditions as well as developing new investigational tools such as blister fluid analysis can help us evaluate this co-presentation of two different immunological processes.

## Data Availability

Not applicable.

## Abbreviations

AGEP: Acute generalized exanthematous pustulosis
TEN: Toxic epidermal necrolysis
TNF: Tumor necrosis factor
NSAIDs: Nonsteroidal anti-inflammatory drugs
